# Potentiation of the Cytotoxic Activity of Copper by Polyphosphate on Biofilm-Producing Bacteria: A Bioinspired Approach

**DOI:** 10.3390/md10112369

**Published:** 2012-10-25

**Authors:** Werner E. G. Müller, Xiaohong Wang, Yue-Wei Guo, Heinz C. Schröder

**Affiliations:** 1 ERC Advanced Investigator Grant Research Group at Institute for Physiological Chemistry, University Medical Center of the Johannes Gutenberg University, Duesbergweg 6, D-55128 Mainz, Germany; Email: wxh0408@hotmail.com (X.W.); hschroed@uni-mainz.de (H.C.S.); 2 National Research Center for Geoanalysis, Chinese Academy of Geological Sciences, 26 Baiwanzhuang Dajie, CN-100037 Beijing, China; 3 State Key Laboratory of Drug Research, Shanghai Institute of Materia Medica, Chinese Academy of Sciences, 555 Zu Chong Zhi Road, Zhangjiang High-Tech Park, CN-201203 Shanghai, China; Email: ywguo@mail.shcnc.ac.cn

**Keywords:** biofilm, marine coatings, antifouling strategies, synergism, polyphosphate, copper, bisphosphonate, bioinspired approach

## Abstract

Adhesion and accumulation of organic molecules represent an ecologically and economically massive problem. Adhesion of organic molecules is followed by microorganisms, unicellular organisms and plants together with their secreted soluble and structure-associated byproducts, which damage unprotected surfaces of submerged marine structures, including ship hulls and heat exchangers of power plants. This is termed biofouling. The search for less toxic anti-biofilm strategies has intensified since the ban of efficient and cost-effective anti-fouling paints, enriched with the organotin compound tributyltin, not least because of our finding of the ubiquitous toxic/pro-apoptotic effects displayed by this compound [1]. Our proposed bio-inspired approach for controlling, suppressing and interfluencing the dynamic biofouling complex uses copper as one component in an alternative anti-fouling system. In order to avoid and overcome the potential resistance against copper acquired by microorganisms we are using the biopolymer polyphosphate (polyP) as a further component. Prior to being functionally active, polyP has to be hydrolyzed to ortho-phosphate which in turn can bind to copper and export the toxic compound out of the cell. It is shown here that inhibition of the hydrolysis of polyP by the bisphosphonate DMDP strongly increases the toxic effect of copper towards the biofilm-producing *Streptococcus mutans *in a synergistic manner. This bisphosphonate not only increases the copper-caused inhibition of cell growth but also of biofilm production by the bacteria. The defensin-related ASABF, a marine toxin produced by the sponge *Suberites domuncula*, caused only an additive inhibitory effect in combination with copper. We conclude that the new strategy, described here, has a superior anti-biofilm potential and can be considered as a novel principle for developing bio-inspired antifouling compounds, or cocktails of different compounds, in the future.

## 1. Introduction

Immediately after submerging a solid phase in freshwater and seawater, biofilm production by microorganisms starts to develop. Biofilm is produced by bacteria to increase the ability of these organisms to survive against external/environmental influences, e.g., extreme temperature, dehydration, UV light, disinfectants, antibiotics or physical stress. In turn, the attaching microorganisms embedded in their secreted material form a specific ecological niche which provides them with an increased resistance against adverse external factors (see [2,3,4]). It had been proposed that the first step in biofilm formation is the adsorption of organic substances and particles from the aqueous environment, a process which results in the assembly of an organic layer onto the surface of the submerged inorganic or organic solid phase [5]. Often bacteria and diatoms have been identified as the first colonizing organisms onto the pre-formed organic layer [6]. The unicellular organisms, more specific their secreted organic matrices (the bacterial biofilm), change not only the topography of the surface of the solid phase but also its (bio)chemical properties providing a secondary platform for other microorganisms including fungi, diatoms, cyanobacteria, microalgae as well as macroalgae and also invertebrates to settle (reviewed in [7,8]). Finally, a biofouling community is formed that is not only complex but also dynamic in their organismic composition and chemical/morphological architecture [9]. Biofouling coatings not only cause health hazards on household and surgical tools but also cause enormous damage to ships and vessels as well as on marine platforms resulting in huge economic losses (see [10,11]). 

The most effective solutions proposed in combatting biofouling on ships and applied throughout the history of navigation has been (1) iron compounds (see [12,13]) and (2) tributyltin (TBT) [14], especially as self-polishing copolymer paints (TBT-SPC paints). Until 10 years ago, 70% of the vessels of the world’s fleet had been protected with TBT [14] resulting in huge economic benefits. Unfortunately, TBT-SPC paints adversely affect the environment. For example, at concentrations of as low as 20 ng/L, TBT causes malformation of shells during growth of the oyster *Crassostrea gigas* and at 1 ng/L imposex of *Nucella* sp. [15,16]. It was our group which succeeded firstly to show that TBT causes apoptosis both in the lowest metazoans, e.g., sponges and in deuterostomian animals [1], and, later, that TBT also causes this effect in protostomians [17]. Based on these and other observed malformations, national regulations in countries all over the world had been released against TBT [18,19]. After the International Convention (5 October 2001), a far-reaching ban of TBT-based antifouling paints had been passed that became effective on 1 January 2008 [20]. Since then intense research programs/projects have been launched [21] to develop efficient substituting anti-biofouling surfaces, e.g., organic/inorganic membranes [22], advanced copper compounds and copper nanoparticles (see [23]) or novel organic bioactive compounds (see [9]), e.g., the bastadins [24] or isoquinoline derivatives [25], all having anti-biofouling activity. 

In a bio-inspired approach we asked the question why some marine organisms, e.g., the sessile-living sponges, are not covered by biofilms resulting in impairment or degradation of their surfaces followed by partial or complete death of the animals. These animals are characterized by the presence of species-specific populations of bacteria [26] which live with the animal host in a symbiosis-related relationship. From this piece of evidence, the conclusion could be deduced that bacteria exist at the surface of the sponges that are producing bioactive compounds which might prevent the formation of a secondary fouling coat by microorganismic biofilms. An experimental support of this attractive assumption has not been presented. We followed a second approach which comes from the observation that sponges, especially demosponges, e.g., *Tethya lyncurium*, are rich in inorganic polyphosphates (polyP) as well as in their catabolic enzymes, the exopolyphosphatases [27] an enzyme that had been identified by us [28]. The polyPs are well established biopolymers, being synthesized both in pro- and eukaryotic organisms [29,30,31] that display antibacterial [32] and also osteoinductive activity [33]. Furthermore, polyP had been described to have anticorrosion properties, e.g., ammonium polyphosphate [34]. 

Experimental data suggest that the bacterium *Acidithiobacillus ferrooxidans* uses polyP as a de-toxification polymer against exposure to high levels of heavy metals, including copper [35]. The data indicated, and documented for the first time, that polyP, after exposure to copper, undergoes increased expression of the exopolyphosphatase. As a result, ortho-phosphate is formed that binds to copper and in turn sequesters the heavy metal out of the cell. In turn, this observation implies that polyP acts as an intracellular storage for ortho-phosphate which binds to copper and detoxifies this metal by exporting it [36]. Also based on these data we test in the present study the hypothesis that polyP might modulate the toxic effect of copper; we used the Gram-positive coccus-shaped bacterium *Streptococcus mutans*, known to be rich in polyP [31] as a model. This bacterium is also known to form bulky biofilm margins [37]. The experimental data given here show that exposure of *S. mutans* towards copper and polyP results in a synergistic toxic effect. Even more interesting, co-incubation of the bacteria with the bisphosphonate DMDP (dichloromethylene diphosphonic acid) causes a stronger synergistic effect together with copper than polyP with copper. Bisphosphonates are pyrophosphate analogs, comprising a carbon atom (P-C-P) instead of an oxygen atom (P-O-P) between the two phosphorus atoms (reviewed in [38]). Bisphosphonates inhibit the resorption of the bone (hydroxyapatite) matrix by osteoclasts (see [39]). The presented experimental data suggest that the bisphosphonate DMDP inhibits the exopolyphosphatase-catalyzed hydrolysis of polyP with the consequence of a higher copper-caused toxicity. In a parallel series of experiments the toxic peptide, the defensin-related ASABF (*Ascaris suum* antibacterial factor), isolated from the sponge *Suberites domuncula*, caused a likewise distinct inhibition of the growth of *S. mutans*. However, this toxic effect had been merely additively in combination with copper. We propose in our bioinspired approach, based on the data given, a model which implies that polyP/bisphosphonate-DMDP together with copper causes *in vitro* an enhanced and synergistically increased inhibition towards the biofilm producing bacteria *S. mutans*, by breaking the natural resistance barrier developed in bacteria against the toxic effect of copper.

## 2. Results

### 2.1. Toxic Effect of Copper on *S. mutans*

**Figure 1:**
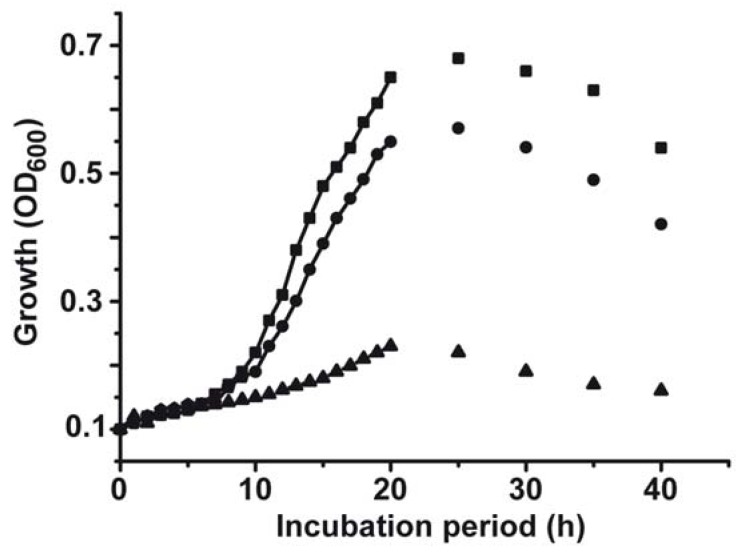
Growth kinetics of *S. mutans* in the absence of any compound (■), in the presence of 3 µM CuSO_4_ (●) or in cultures with 100 µM CuSO_4_ (▲). The growth of the bacteria was monitored optically at 600 nm.

**Figure 2:**
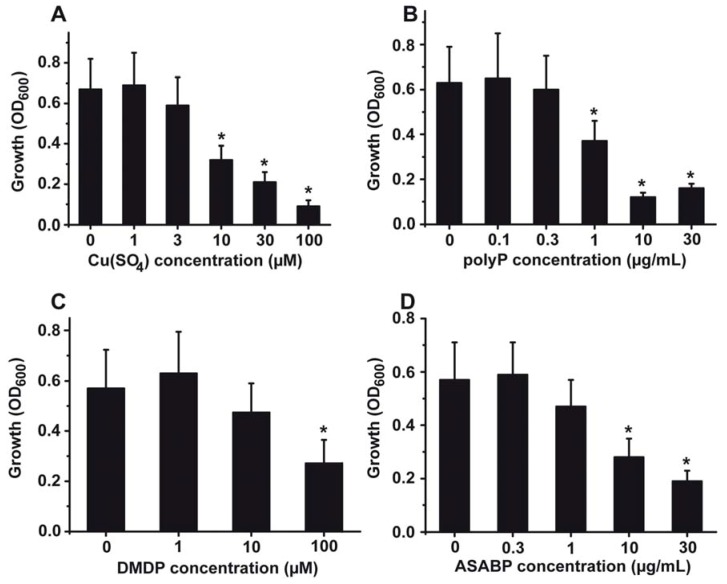
Growth inhibitory effect of the inhibitory compounds used in this study, added alone to the cultures: (**A**) CuSO_4_, (**B**) biopolymer polyphosphate (polyP), (**C**) bisphosphonate dichloromethylene diphosphonic acid (DMDP) and (**D**) the marine toxin (the defensin-related *Ascaris suum* antibacterial factor (ASABF)) produced by the sponge *S. domuncula*. The results are expressed as means (10 experiments each) ± SD of the mean; * *P* < 0.01.

The effect of copper on the growth rate of *S. mutans* was studied *in vitro* in liquid culture medium. The evaluation of the growth rate was determined electronically, as described under “Experimental Section”. The growth kinetics revealed that after a lag phase of 10–12 h the logarithmic growth starts; the plateau phase is reached after 20–25 h (Figure 1). After 30 h the density of the bacteria drops again. After addition of copper(II) sulphate (CuSO_4_) at a concentration of 3 µM the extent of growth becomes impaired, by 18%. Addition of 100 µM CuSO_4_ results in an inhibition of growth by 82%. A detailed dose response relationship is given in Figure 2A. The bacteria were grown for 20 h; after that period the optical density was measured for each culture. A significant drop of the growth, as measured on the basis of the recorded OD_600_ units, is seen at 10 µM CuSO_4_ with 0.32 ± 0.07 OD_600_ units compared to the controls with 0.67 ± 0.15 OD_600_ units (Figure 2A). At higher concentrations the copper-caused effect is more pronounced.

### 2.2. Effect of PolyP on the Gram-Positive *S. mutans*

It had been generalized that Gram-positive bacteria, and in turn also *S. mutans*, are affected by polyP (see [31,40]). The antibacterial effect on *S. mutans* is significant at a concentration of 1 µg/mL (Figure 2B). At this concentration a 38% growth inhibition is measured. At higher concentrations (e.g., 10 µg/mL) the inhibitory effect is even more pronounced (reduction by 74%). Similar effects are seen in the system with *Porphyromonas gingivalis* [40]. 

### 2.3. Effect of Bisphosphonates

The effect of the bisphosphonate DMDP on *S. mutans* is comparably lower. Within the concentration range of 1–10 µM DMDP does not display any significant toxic effect, while at the concentration of 100 µM a significant antibacterial effect is seen; namely a reduction of the cell density after an incubation period of 20 h from 0.57 ± 0.15 to 0.26 ± 0.08 OD_600_ units (Figure 2C). 

### 2.4. The Inhibitory Activity of the Antimicrobial Peptide from *S. domuncula*

The gene encoding the ASABF-type antimicrobial peptide, identified in the sponge *S. domuncula*, had been cloned and subsequently expressed in a recombinant manner [41]. In this earlier study it had been reported that ASABF peptide inhibits Gram-positive bacteria within a concentration range (MIC) of 1 to 5 μg/mL. In the present study, a significant antibacterial activity of the ASABF-type peptide towards *S. mutans* was seen at concentrations above 10 µg/mL, as shown in Figure 2D.

### 2.5. Evaluation of the Combinatorial Potential of Copper Together with the Other Antimicrobial Agents Against *S. mutans* Growth

**Figure 3:**
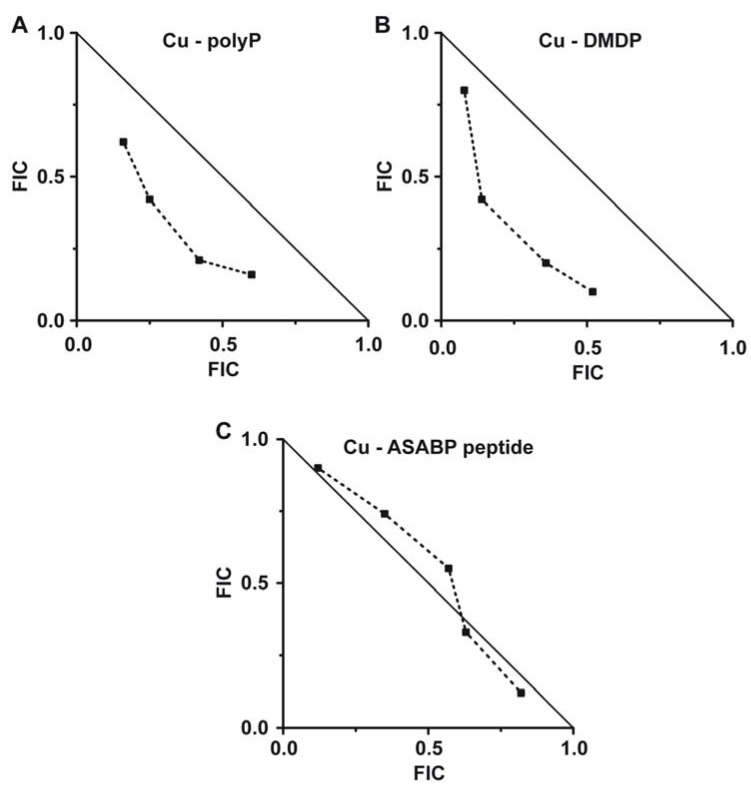
Isobolograms showing the synergistic effect (**A**) of polyP and (**B**) of the bisphosphonate DMDP on copper-caused toxicity on *S. mutans*. (**C**) The marine toxin (defensin-related ASABF) from *S. domuncula* caused only an additive effect on copper toxicity.

The determination of the fractional inhibitory concentration (FIC) is an informative measure for a potential synergistic/antagonistic/additive effect of two compounds, given in combination [42]. In the present study we determined that the FIC index for the antimicrobial compound combination between copper and polyP is 0.55, indicative of a synergistic effect of the two compounds (Figure 3A). Moreover, if copper is administered together with the bisphosphonate DMDP a very low FIC index of only 0.48 is calculated (Figure 3B). This latter result strongly indicates that the bisphosphonate DMDP acts highly inhibitory and highly synergistic with copper on *S. mutans*. In contrast, the FIC index copper to ASABF-type peptide was found to be ≈0.5 indicating that the peptide acts only additive to the antibacterial function of copper (Figure 3C). 

### 2.6. Effect of Copper on PolyP Content in *S. mutans*

**Figure 4:**
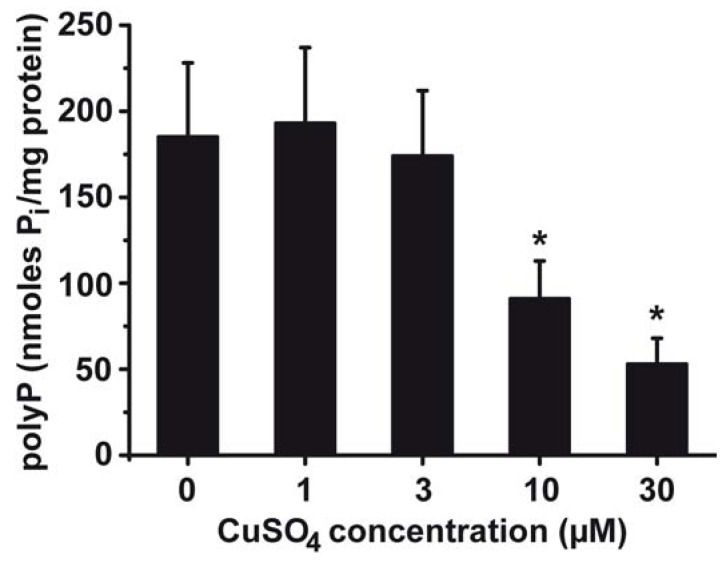
Effect of CuSO_4_ in the culture medium of *S. mutans *on the intracellular polyP content. The cells were exposed to different concentrations of CuSO_4_ for 25 h. Then the cells were broken and assayed for polyP level using the two-step enzymatic conversion of polyP into ATP. The polyP level is correlated with the bacterial protein content from which polyP was quantitatively determined. Data are means (6 experiments each) ± SD of the mean; * *P* < 0.01.

From previous studies it is known that in some bacteria, polyP is induced after exposure to xenobiotics (see [31,43]). To clarify whether *S. mutans* can also adapt its polyP level towards the environment exposure, studies with increasing concentrations of CuSO_4_ were performed (Figure 4). After incubation of the bacteria for 25 h, in the absence of copper, the level of polyP in the cells was determined to be 184 ± 43 nmoles P_i_/mg bacterial protein. Addition of 1 or 3 µM CuSO_4_ does not significantly change that level. However, in cultures with 10 or 30 µM CuSO_4_ the level of intracellular polyP drops to 91 ± 22 or to 53 ± 15 nmoles P_i_/mg, respectively. 

### 2.7. Phosphate (P_i_) Efflux from *S. mutans*

**Figure 5:**
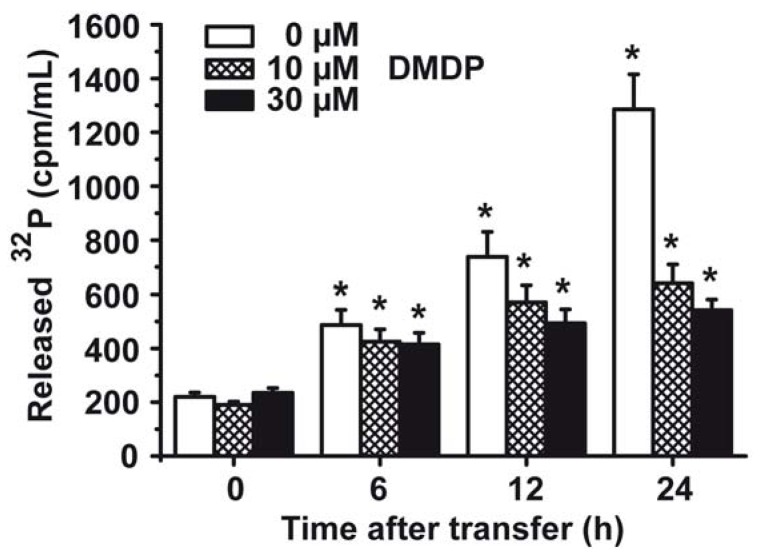
Efflux of P_i_ from* S. mutans *during incubation with the bisphosphonate DMDP. At first the cells were labeled with H_3_[^32^P]O_4_ and then transferred to medium without H_3_[^32^P]O_4_. Then the cells were continued to be incubated either in the absence (0 µM) or presence (10 µM or 30 µM) of DMDP for 0 to 24 h, as indicated. At the time indicated the cell free supernatants were collected by centrifugation and counted for radioactivity. Means (6 experiments each) ± SD are given; * *P* < 0.01.

S. *mutans* cells were labeled H_3_[^32^P]O_4_ during growth in tryptone-vitamin based medium. After transfer to medium, lacking H_3_[^32^P]O_4_, the cells were continued to be cultured. Aliquots were taken, centrifuged and the radioactivity in the supernatant was collected for determination of ^32^P, and in turn for [P_i_] release. The data summarized in Figure 5 show that cells not treated with the bisphosphonate DMDP release significantly more ^32^P into the culture medium compared to cells that had been incubated with 10 µM DMDP or 30 µM DMDP, respectively. During a 24 h incubation of the cells, after pulsing with H_3_[^32^P]O_4_, the controls released 1285 ± 131 cpm/mL, compared to 639 ± 71 cpm/mL (10 µM DMDP), or 542 ± 39 cpm/mL (30 µM DMDP), respectively. 

### 2.8. Inhibition of Biofilm Production

**Figure 6:**
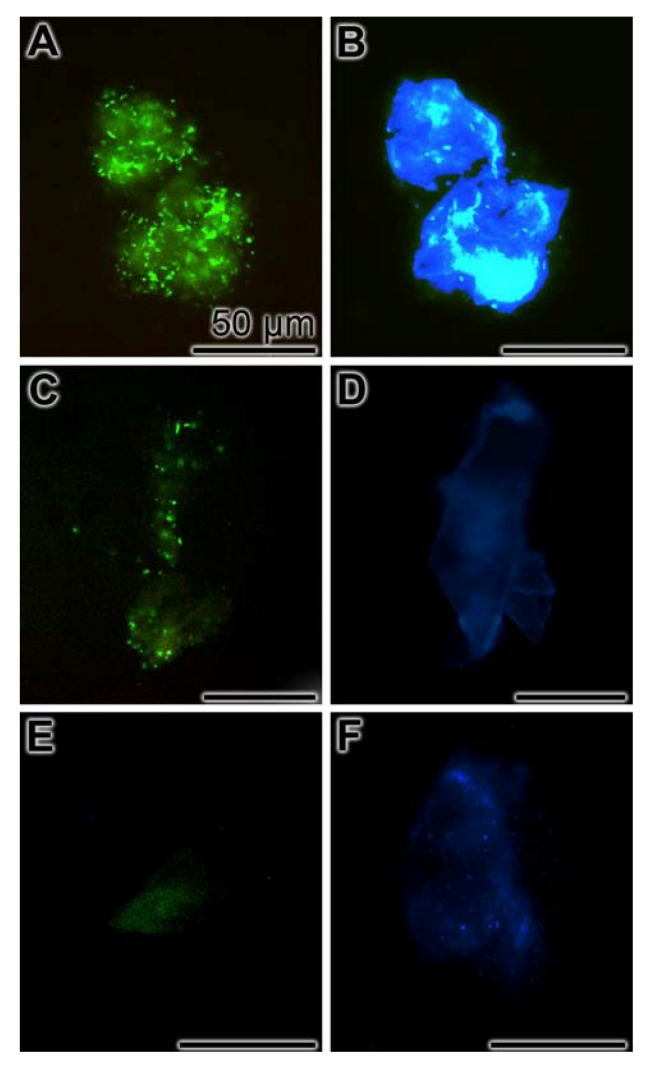
Inhibition of biofilm production by polyP/bisphosphonate-DMDP. *S. mutans* had been grown on microscope slides for 5 days (**A** and **B**) in the absence of any inhibitory compound, in the presence of 1 µg/mL of polyP (**C** and **D**), or with 1 µg/mL of polyP together with 1 µg/mL bisphosphonate-DMDP (**E** and **F**). After incubation the specimens were reacted with WGA-labeled with Alexa Fluor 555 and DAPI. Then the samples were analyzed for fluorescence of the labeled WGA-lectin (**A**, **C**, **E**), or for DAPI (**B**, **D**, **F**).

S. *mutans*, if growing as biofilms, bind with the sialic acid termini, existing at their glycoproteins/polysaccharides, to the WGA-lectin [44,45]. Using this lectin as a tool, under the conditions used, a bright fluorescence was seen at the bacterial plaques formed by *S. mutans* in the absence of any additional inhibitory component (Figure 6A). In parallel, the bacterial colonies were visualized by DAPI (Figure 6B). However, if the bacteria growing onto the microscope slides were exposed for 5 days to 1 µg/mL of polyP a significant reduction of the staining intensity due to WGA-labeled with Alexa Fluor 555 was seen (Figure 6C). The DAPI stained colonies are shown in parallel (Figure 6D). There was a very strong reduction of the labeled WGA to the bacteria in cultures that had been incubated with 1 µg/mL of polyP together with 1 µg/mL of bisphosphonate DMDP (Figure 6E); parallel image, stained with DAPI (Figure 6F).

## 3. Discussion

The initial phases of the biofouling process are fast; within seconds to minutes an organic layer is formed by adsorption of organic matter from the environment followed by a settlement of microorganisms which takes place within minutes (primary community). After formation of that biofilm layer, secondary organisms, macrofoulers (spores of algae and larvae of animals, e.g., worms or barnacles) are attaching, a process that requires hours to days (secondary community). Finally, algae, sponges and worms (tertiary community) interact and start to grow on the layers of the primary and secondary communities (reviewed in [8]). In this complex community a selection pressure for the most suitable living conditions of a given taxon causes a change of composition of all three levels of communities. The driving forces controlling the speed and the direction of the proceeding population dynamics are (i) the quorum sensing system coordinating the interactions of the microorganisms by the release of stimuli and the response to them within a population [46] and (*ii*) the formation of resistance barriers formed by each taxon. The resistance mechanism by which multicellular animal species can cooptate to their suitable environment filled both with favorable and adverse signals is the P-glycoprotein P-170, also known as a multidrug-resistance transporter, that extrudes xenobiotics out of the cells under ATP consumption (reviewed in [47]). The relevance of the P-170 system as a survival and decay decision-making factor for a given marine/freshwater animal species had been elucidated by us [48]. It had been established that the P-170 mechanism controls the viability and population response of the clam *Corbicula fluminea* to aromatic amines [49] or to other xenobiotics, e.g., 2-acetylaminofluorene or vincristine and daunomycin. This transporter can act both as a positively and as a negatively regulatory machinery for the natural resistance of aquatic organisms [50]. To mention is here that the P-170 system of marine animals is strongly impaired by toxins formed by biofilm-producing microorganisms, “marine snow”, and results in mass killing of e.g., sponges, mussels, or Anthozoa [51]. 

Focusing on the resistance/stimulation quorum sensing system, signals are released that change the gene-expression and phenotypic response of the neighboring microorganism [52]. In the present study experimental evidence is given that microorganisms can also adapt to an environmental adverse condition without using quorum sensing signals but using their own intracellular resistance machinery, e.g., the polyP system. Even more, bacterial mutants lacking the polyP synthesizing machinery are defective in quorum sensing and biofilm formation [53]. 

As outlined in the “Introduction” copper compounds are potent anti-biofouling agents widely used to protect the surfaces of ships against biofilm production, followed by biofouling (reviewed in [23]). However, those biofilm-producing bacteria acquire resistance against (heavy) metals, e.g., against iron [54] or copper [55]. Hence an economically preferred and functionally indicated solution for an efficient copper-based anti-biofouling protection of the surfaces of solid phases, e.g., the outer surface of ship hulls, would be to develop bioactive compounds/polymers that can be embedded in the copper-containing paints used for protection. More specifically, the second component should result in an increased sensitization of the attaching microorganisms onto the copper-coated surfaces. This prerequisite is fulfilled if the second component co-exists with copper in the paint to be active simultaneously with copper on the solid phase. To test the feasibility of such a much-needed solution to improve the anti-biofouling action of copper-coated surfaces we used the biofilm-producing, Gram-positive bacterium *S. mutans*. The experiments revealed that *S. mutans* is sensitively inhibited by copper. 

In the present study we used CuSO_4_ as a copper compound. Copper (Cu) as a transitional metal occurs in nature in several oxidation states: Elemental copper Cu(0) (solid metal), Cu(I) cuprous ion, Cu(II) cupric ion, and rarely Cu(III) [56]. The most prevalent form of copper in the aqueous milieu is Cu^2+^ [56]. Besides being required as a co-factor for a series of enzymes or functional proteins [23], copper displays adverse effects, e.g., accumulation in distinct tissues resulting in the formation of cirrhosis/fibrosis and hyperplasia, followed by lysosomal damage, apoptotic and necrotic cell death (see [57]). CuSO_4_ in the *S. mutans* system caused a 50% cell death at a concentration of approximately 10 µM, matching with previous data using mammalian cells [57]. Copper(I) oxide (Cu_2_O) is slightly more toxic with around 5–10 µM towards marine microalgae [58]. The inhibitor effect of polyP, added to the bacteria in the culture medium, is comparable with a 50% inhibitory dose of around 1 µg/mL, as shown here. Furthermore, almost no inhibitory effect is seen with bacteria that are exposed to the synthetic pyrophosphate-analogue, the bisphosphonate DMDP.

The general mode of action of polyP on microorganism has been well studied [31,43]. This polymer is essential for a series of metabolic (intermediary metabolism) pathways in bacteria and animals, as well as for growth control. In addition, polyP can attach to cell membranes, and as a polyanion can complex Ca^2+^ out of the cell membrane, followed by a lysis of the cells [59]. For some microorganisms, e.g., for *Bacillus subtilis*, it has been proposed that polyP can be taken up if complexed with biological polymers (poly-β-hydroxybutyrate), after salvation through a pump like system [60]. As outlined in the “Introduction” polyP might function as a deposit for ortho-phosphate that forms salts with copper, allowing and facilitating the heavy metal to be exported/sequestered from the microorganisms. Ortho-Phosphate is released from the polymer after hydrolysis by exopolyphosphatase [35]. To test this assumption the *S. mutans* were tested with the pyrophosphate analogue, the bisphosphonate DMDP, to interfere with the hydrolysis of polyP. Measuring the extent and rate of ortho-phosphate export in the presence of the bisphosphonate DMDP it could be demonstrated that this analogue drastically reduces the release of ortho-phosphate from the cells. This finding is taken as evidence that DMDP causes an inhibition of the intracellular hydrolysis reaction of polyP, very likely via an inhibition of the exopolyphosphatase. If this assumption indeed reflects the reaction *in vivo*, growth inhibition studies of the bisphosphonate DMDP together with copper should be performed. Under the assumption that (*i*) polyP exists in the Gram-positive *S. mutans*, which has been determined in the present study, and (*ii*) that the bisphosphonate DMDP causes a synergistic effect on the toxicity of copper, the most substantiated conclusion is that the bisphosphonate inhibits the intracellular hydrolysis of polyP to ortho-phosphate. 

In a final series of experiments the effect of polyP on the capacity of biofilm formation was studied. The data revealed that in the presence of polyP (1 µg/mL) the biofilm synthesis is substantially inhibited during the 5 days incubation period, while exposure of the bacteria to polyP (1 µg/mL) and DMDP (1 µg/mL) results in an (almost) complete inhibition. 

While the inhibitory activity of polyP and of the bisphosphonate was found to be synergistic with the growth reducing effect of copper, the influence of the sponge-derived anti-microbial ASABF-type antimicrobial peptide was found to be additive to the potency of copper. The latter result is taken as evidence that the mode of action of the ASABF peptide is different from the one of copper. From the analyses of the biological activity of the ASABF peptide it had been shown that the peptide lyses both mammalian and sponge cells [41]. The ASABF peptide had been included in the present study since this compound is accumulated within the surface layer of the sponge, suggesting an antifouling activity in the *S. domuncula* model system. 

## 4. Experimental Section

### 4.1. Materials

Na-polyP (average chain of approximately 40 phosphate units) was obtained from Chemische Fabrik Budenheim (Budenheim, Germany); the bisphosphonate dichloromethylene diphosphonic acid disodium salt (Clodronate disodium salt, DMDP, product No. D4434) and 4′,6-diamidino-2-phenylindole (DAPI) from Sigma (Taufkirchen, Germany); H_3_[^32^P]O_4_ (≈120 Ci/mL) from OurChemical (Futian District, Shenzhen, China); wheat germ agglutinin/lectin-Alexa Fluor 555 conjugate from Molecular Probes (Eugene, OR, USA).

### 4.2. Antimicrobial Peptide from *S. domuncula*

The ASABF-type antimicrobial peptide, produced by the sponge *S. domuncula* was prepared in a recombinant manner as described recently [41]. 

### 4.3. *S. mutans* and Culture Condition

*S. mutans* strain ATCC 25175, obtained from DSMZ-German Resource Centre for Biological Material (Braunschweig, Germany) was cultivated on 5% defibrinated sheep blood agar as described [61]. Incubation was performed at 37 °C in an incubator (5% CO_2_). As antibiotic erythromycin (10 µg/mL) was used. The growth kinetics and the inhibition studies were performed in the tryptone-vitamin based medium [62] that had been supplemented with 0.5% D(+)-glucose. The growth kinetics were monitored electronically (Bioscreen C reader, Labsystems, Helsinki, Sweden) at 600 nm (OD_600_) every 60 min. 

### 4.4. Inhibition Studies

PolyP was added as Ca^2+^ salt, complexed by addition of the Na-salt of polyP with Ca^2+^ at a stoichiometric molar ratio of 2:1 [63].

### 4.5. Evaluation of the Combinatorial Potential of Copper Together with the Other Antimicrobial Agents Against *S. mutans* Growth

To assess the activity of polyP, the bisphosphonate DMDP, and the ASABF-type peptide on copper in combination experiments, dose-response experiments were performed with (*i*) copper alone (around the ED_50_ inhibitory concentration), (*ii*) with the second compound at sub-inhibitory concentrations and (*iii*) in combinations (at least four concentrations each) of the of two agents. From the growth curves the ED_50_ inhibitory concentration was assessed by logit regression [64]. The ED_50_ concentration was chosen to calculate the “fractional inhibitory concentration” (FIC) as described [42,65,66]. Subsequently, the FIC values of each pair of combination were plotted. The FIC index of the antibacterial effect of copper for the three combinations is indicative for the combinatory antibacterial effect; if the FIC index (FIC_A_ + FIC_B_) is equal to 1.0, an additive effect exists; if the FIC index is <1, the effect is synergistic, and if the index is >1, and antagonistic effect has to be deduced. 

### 4.6. Cultivation of *S. mutans* on Microscope Slides for Biofilm Formation

S. *mutans* was cultivated in liquid broth. After growth for overnight 300 µL aliquots were removed and placed onto a microscope slide. After allowing the cells to adhere to these surfaces (3 h) the slides were placed into petri dishes and covered with tryptone-vitamin based medium, enriched with 0.5% D(+)-glucose. The cultures were then incubated for 5 days. The samples were carefully washed with PBS and then reacted with the lectin wheat germ agglutinin (WGA), fluorescently labeled with Alexa Fluor 555, as described [45]. In parallel, the specimens were stained with 4′,6-diamidino-2-phenylindole (DAPI). The slides were inspected with an Olympus AHBT3 microscope under immunofluorescence light suitable for WGA, fluorescently labeled with Alexa-stained structures or for DAPI.

### 4.7. Quantification of PolyP

S. *mutans* cultures, grown to the stationary phase (after 25 h) were extracted after lysis of the cells with guanidinium isothiocyanate, followed by binding to silicate glass, as described [67]. Then polyP was determined by two step enzyme assay. In doing so, polyP was first converted into ATP by the polyP kinase, and then ATP was measured by the firefly luciferase ATP assay [35,67]. The luminescence was determined with a luminometer, detecting emitted light between 390 and 620 nm [68]. The concentration of polyP was calculated after setting-up a standard curve for ATP [67]. The values were correlated to the protein content in the samples, used for polyP determination.

### 4.8. Phosphate Efflux Determinations

S. *mutans* cells were labeled by growing them in tryptone-vitamin based medium, supplemented with 0.5% D(+)-glucose for 25 h. After the 25 h incubation period, the cultures were centrifuged and re-suspended at a density of 10^10^ cells/mL in medium enriched with 0.1 mM ortho-phosphate, essentially as described [35]. H_3_[^32^P]O_4_ (≈120 Ci/mL) was added and the cultures were continued to be incubated for 12 h. Then the cells were washed, transferred to unlabeled standard medium, supplemented with 0, 10 or 30 µM DMDP, and continued to be incubated for 24 h. Aliquots were taken at the indicated periods of time, centrifuged and the radioactivity was determined in the cell-free medium using liquid scintillation spectrometry (Top-Count, Packard Instrument, Meriden, CT, USA). 

### 4.9. Further Methods

The results were statistically evaluated using the paired Student’s *t*-test [64]. For quantification of protein the Bradford method (Roti-Quant solution—Roth) was used [69].

## 5. Conclusions

**Figure 7:**
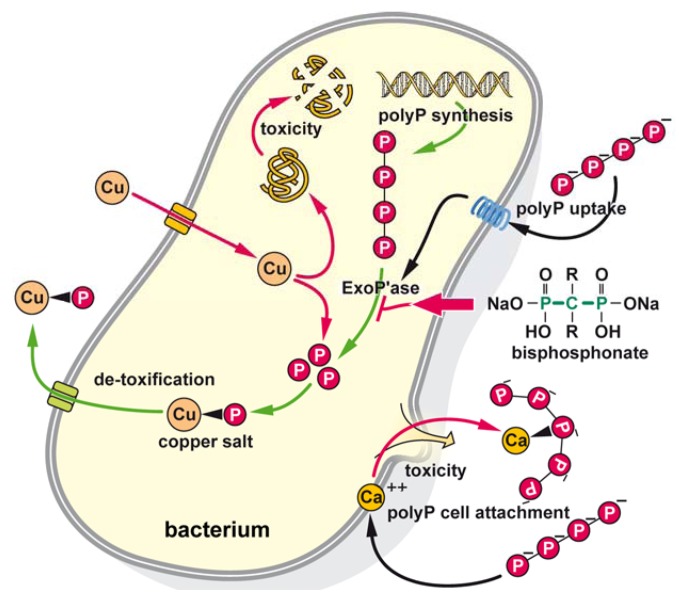
Schematic outline of the synergistic effect caused by copper in combination with polyP/bisphosphonate on bacteria cells. This bioinspired concept implies that copper (Cu), taken up from the solid phase onto which the bacteria are in the process to attach, is imported into the cells and displays it toxicity. In a parallel de-toxification process polyP that is either formed intracellularly or taken up by the polyP-3-hydroxybutyrate-Ca^2+^ pump undergoes enzymatic hydrolysis by the exopolyphosphatases (ExoP’ase) under formation of ortho-phosphate. In turn ortho-phosphate forms with copper an intracellular salt that is exported from the bacterial cells. In a parallel mode of action polyP, extracellularly attaching to the bacterial membranes removes Ca^2+^ from the cell membrane and by that causes death of the microorganisms.

Based on the data presented here it can be concluded that in bacteria polyP, after enzymatic hydrolysis to ortho-phosphate, binds to influxed copper ions which are co-transported as a phosphate salt out of the cell. It is highlighted that bisphosphonate inhibits the enzymatically (exopolyphosphatase) mediated hydrolysis of polyP and in turn prevents the ortho-phosphate mediated detoxification of the heavy metal ion. PolyP is either formed intracellularly or imported into the cells via a postulated polyP-3-hydroxybutyrate-Ca^2+^ pump (see [31]). In a second mode of action, polyP chelates out of the bacterial cell membrane Ca^2+^ with the consequence of a permeabilization of the cell membrane, followed by cell death. A schematic summary is given in Figure 7. Future studies must clarify if polyP displays any effect on marine macro-foulers, e.g., algae and barnacles. Their incorporation in coatings via formulation represents a great challenge. In addition, techniques must be designed to incorporate in a stable way polyP into a paint that can be applied onto the surface of a ship hull.

After having established the polyP-based detoxification pathway, which is likely to exist also in bacteria in the field, it can be expected that new anti-biofouling strategies for the formulation of paints to be used to protect hulls against biofilm-producing microorganisms can be developed. The advantage of such a strategy would be that only the attaching organisms are co-affected by the two active principles, copper and polyP, while the non-attached organisms remain non-influenced. These two compounds act synergistically, implying that lower concentrations of the individual bioactive components are required to be included into the paint.
